# The impact of susceptibility correction on diffusion metrics in adolescents

**DOI:** 10.1007/s00247-021-05000-3

**Published:** 2021-04-24

**Authors:** Katri Lahti, Riitta Parkkola, Päivi Jääsaari, Leena Haataja, Virva Saunavaara, On behalf of the PIPARI Study Group, Annarilla Ahtola, Mikael Ekblad, Satu Ekblad, Eeva Ekholm, Linda Grönroos, Leena Haataja, Mira Huhtala, Jere Jaakkola, Eveliina Joensuu, Max Karukivi, Pentti Kero, Riikka Korja, Katri Lahti, Helena Lapinleimu, Liisa Lehtonen, Tuomo Lehtonen, Marika Leppänen, Annika Lind, Hanna Manninen, Mari Koivisto, Mira Mattson, Jonna Maunu, Petriina Munck, Laura Määttänen, Pekka Niemi, Anna Nyman, Pertti Palo, Riitta Parkkola, Liisi Ripatti, Päivi Rautava, Katriina Saarinen, Tiina Saarinen, Virva Saunavaara, Sirkku Setänen, Matti Sillanpää, Suvi Stolt, Päivi Tuomikoski-Koiranen, Timo Tuovinen, Karoliina Uusitalo, Anniina Väliaho, Milla Ylijoki

**Affiliations:** 1 Department of Pediatric Neurology, University of Turku and Turku University Hospital, P.O. Box 52, 20521 Turku, Finland; 2 Department of Adolescent Psychiatry, Turku University Hospital, Turku, Finland; 3 Department of Radiology, University of Turku and Turku University Hospital, Turku, Finland; 4 Department of Oral and Maxillofacial Diseases, Turku University Hospital, Turku, Finland; 5 Children’s Hospital, and Pediatric Research Center, University of Helsinki and Helsinki University Hospital, Helsinki, Finland; 6 Department of Medical Physics, Turku University Hospital, Turku, Finland; 7 Turku PET Centre, Turku University Hospital, Turku, Finland

**Keywords:** Adolescents, Brain, Diffusion tensor imaging, Magnetic resonance imaging, Susceptibility correction, Tractography

## Abstract

**Background:**

Diffusion tensor imaging is a widely used imaging method of brain white matter, but it is prone to imaging artifacts. The data corrections can affect the measured values.

**Objective:**

To explore the impact of susceptibility correction on diffusion metrics.

**Materials and methods:**

A cohort of 27 healthy adolescents (18 boys, 9 girls, mean age 12.7 years) underwent 3-T MRI, and we collected two diffusion data sets (anterior–posterior). The data were processed both with and without susceptibility artifact correction. We derived fractional anisotropy, mean diffusivity and histogram data of fiber length distribution from both the corrected and uncorrected data, which were collected from the corpus callosum, corticospinal tract and cingulum bilaterally.

**Results:**

Fractional anisotropy and mean diffusivity values significantly differed when comparing the pathways in all measured tracts. The fractional anisotropy values were lower and the mean diffusivity values higher in the susceptibility-corrected data than in the uncorrected data. We found a significant difference in total tract length in the corpus callosum and the corticospinal tract.

**Conclusion:**

This study indicates that susceptibility correction has a significant effect on measured fractional anisotropy, and on mean diffusivity values and tract lengths. To receive reliable and comparable results, the correction should be used systematically.

## Introduction

Diffusion tensor imaging and diffusion-tensor-based tractography are used in modern neuroscience to study brain white matter [1–3]. Diffusion imaging is widely used in minors to study neurodevelopment, white matter processes, and adversities preceding neurologic or psychiatric diseases [3–6]. The diffusion properties of white matter are affected by myelination and axonal features, for example, but the metrics are also influenced by the tract volume and iron and water content inside a voxel [3, 7].

Diffusion tensor imaging is prone to image artifacts such as distortion, signal loss and blurring, Nyquist ghosts and chemical shift artifacts. Image distortions and signal loss are caused by magnetic susceptibility variations, eddy currents, B0-field inhomogeneities and concomitant magnetic field artifacts [8]. Of these, eddy current artifacts are the most widely studied, but susceptibility artifacts are also known to affect imaging protocols [9, 10].

The term susceptibility artifact refers to a visual distortion consisting of signal loss and pile-up, caused by local alterations in the magnetic field [11]. Alterations occur locally, near junctions between two tissues with different magnetic susceptibilities, e.g., tissue–air and soft-tissue–bone interfaces. This includes the region of the paranasal sinuses and temporal bones and the regions near the spinal canal opening, the cerebellum and the base of the skull. The artifact tends to degrade the phase coherence, especially in the frontal lobe [12–14]. Susceptibility artifacts can cause severe voxel shifts and deviate the image volumes from the subject’s true anatomy to a clinically significant extent [15, 16]. This leads to errors in tensor calculation and, consequently, in diffusion metrics and tractography [16–18].

It has been suggested that susceptibility distortions could be diminished by adjusting imaging parameters [13, 19]. Artifacts can be corrected by using geometric corrections of the structural image [20, 21], estimate maps of B0 inhomogeneities acquired using gradient echo scans [17, 22], and estimates of the underlying distortions derived from additional data that are acquired using different phase-encoding [17, 19, 23]. The use of reversed phase-encoding has been shown to be a reliable method for correcting geometric distortions and recovering lost data [24]. The aim of this study was to explore the impact of susceptibility correction on diffusion metrics in adolescents.

## Materials and methods

### Participants

This study included 82 adolescents born at Turku University Hospital in 2003. The participants were healthy full-term controls (i.e. gestational age ≥37 weeks) from a larger longitudinal cohort study called PIPARI — Development and Functioning of Very Low Birth Weight Infants from Infancy to School Age — and they were recruited at the maternity ward at the time of birth. The recruitment protocol is described in detail in the work of Munck et al. [25]. Of the original 82 subjects, 52 did not participate in this imaging study. The reasons were that the adolescent refused to participate in this imaging study or the parents withdrew the child from the study in an earlier phase of this longitudinal follow-up.

A group of 30 adolescents met the inclusion criteria. Two of these were excluded because of failed MRI and one because of incidental findings in frontal white matter. The mean age of the 27 remaining adolescents (18 boys, 9 girls) was 12.7 years (standard deviation [SD] 0.27 years, range 12.1–13.1 years). None of the participants was diagnosed with psychiatric or neurologic conditions or was receiving psychotropic/neurologic medication at the time of scanning.

The study protocol was approved by the ethics review committee of the Hospital District of Southwest Finland in 2012. At the age of 13, the adolescents and their parents provided separate consents. Fixed orthodontic appliances, including arch wires, palatal or lingual arches and molar bands, were removed before and replaced after the scan for patient security reasons and to minimize ferromagnetism-related artifacts.

### Magnetic resonance imaging

The imaging was performed using a 3-tesla (T) Ingenuity TF positron emission tomography (PET)/MR scanner (Philips Healthcare, Amsterdam, the Netherlands). A SENSE (sensitivity encoding) Head 32-channel coil was used (Philips). The basic anatomical sequences and imaging parameters used in this study are shown in Table 1.

**Table 1:** Parameters of the basic anatomical imaging sequences

Sequence	3-D T1 turbo field echo	T2-weighted turbo spin echo	Fluid-attenuated inversion recovery
Orientation	Sagittal	Transversal	Coronal
Field of view (mm × mm)	256×265	230×179.8	203×183
Voxel size (mm × mm)	1×1	0.45×0.45	0.45×0.45
Slice thickness (mm)	1	3	4
Parallel factor	2	–	–
Repetition time (ms)	8.1	3,000–5,000	10,000
Echo time (ms)	3.7	80	125
Flip angle	7°	–	–
Inversion delay (ms)	–	–	2,800
Duration	4 min 23 s	2 min 7 s	3 min 30 s

*min* minutes, *s* seconds

Two diffusion tensor data sets were collected. The first axial diffusion tensor imaging was performed using a spin-echo echoplanar sequence with a 2-mm slice thickness. There was no gap between slices. Field of view was 256×256 mm with a 128×128 matrix. Reconstruction voxel size was 2×2 mm. A total of 80 slices were collected. Data were collected using 63 directions with a b value of 1,000 and one with a b value of 0. The repetition time was 9,950 ms and the echo time was 90 ms. Parallel imaging factor 3 was used. The oversampling factor in the phase-encoding direction was 1.5. Data were collected with an anterior–posterior fold over direction, and the fat shift direction was posterior. Bandwidth in the echoplanar imaging frequency direction was 1,786.9 Hz. Fat suppression was done using spectral presaturation with inversion recovery. Sequence duration was 10 min 56 s. The second diffusion tensor imaging sequence was collected with similar imaging parameters, except that the fat shift direction was anterior and the data were collected using only six directions with a b value of 1,000 and one with a b value of 0. Sequence duration was 30 s.

### Quality control and data analysis of the diffusion data

We carried out data quality control using DTIPrep [26]. We removed volumes with intensity artifacts, such as severe signal loss, from the data of each subject. The data were corrected for eddy current and motion artifacts. Data were accepted to the study if 30 or more volumes were of acceptable quality [27]. Volume count varied from 39 to 62. Images with a b value of 0 were not included in the automatic quality control protocol, but they were visually inspected for both diffusion tensor sequences.

**Fig. 1:**
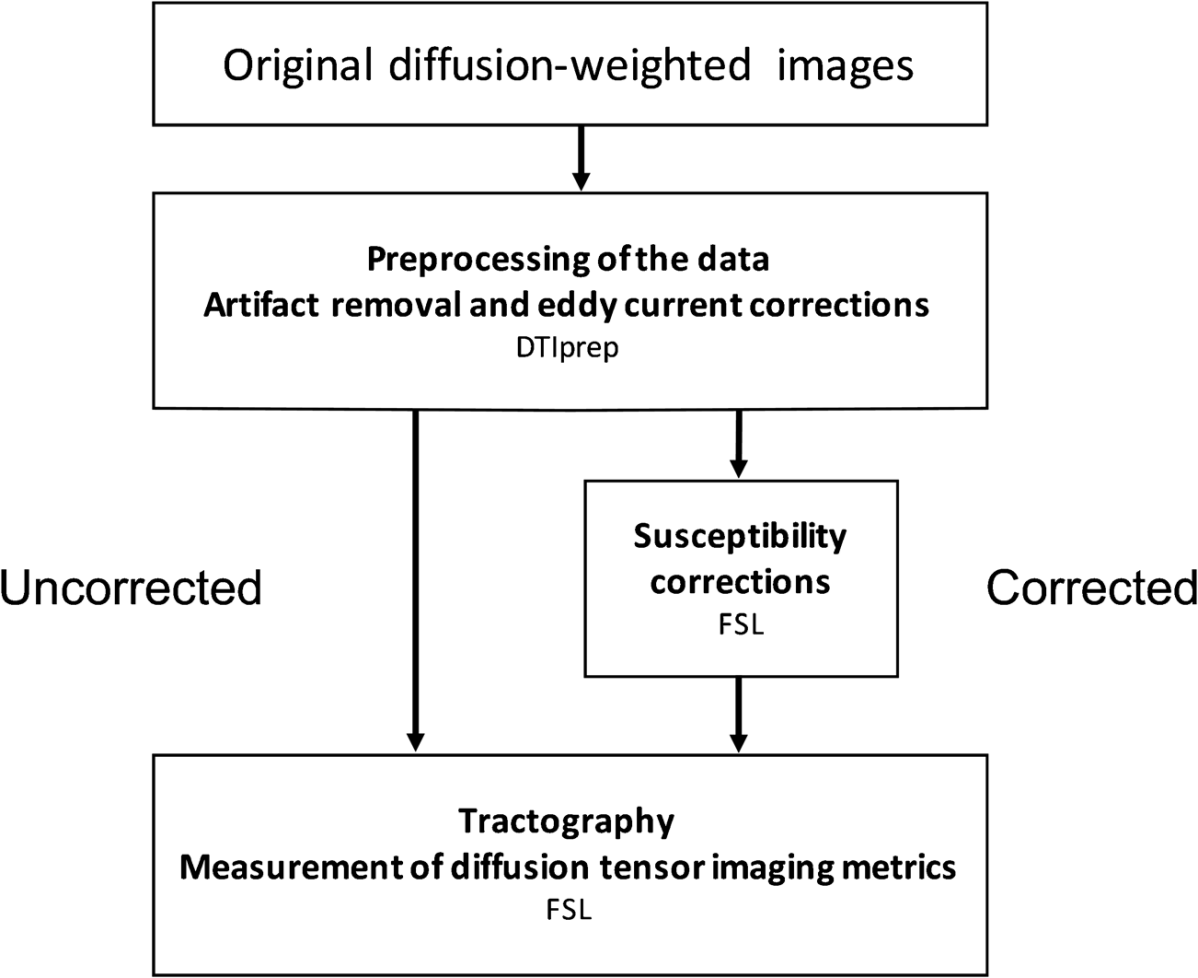
Corrected and uncorrected analysis pathways. *FSL* FMRIB Software Library

After DTIPrep, the data were corrected for susceptibility artifacts using top-up technique [19, 28] of the Functional Magnetic Resonance Imaging of the Brain (FMRIB) Software Library v 5.0.7 [29]. Brain extraction was performed [30]. We calculated the fractional anisotropy and mean diffusivity maps by fitting a tensor model to the raw diffusion data using FMRIB’s Diffusion Toolbox. We calculated parametric maps for cases both with and without susceptibility corrections. The analysis pathways are shown in Fig. 1. We visually inspected the main eigenvector’s direction using FslView in three structures: the corpus callosum (left–right), corticospinal tract (cranio–caudal) and cingulum (anterior–posterior).

### Data analysis of the anatomical data

We analyzed the anatomical data using FreeSurfer version 5.3.0 [31, 32]. Operator K.L., with 3 years of experience in pediatric neuroradiology (under the supervision of R.P., professor in neuroradiology), visually inspected and manually corrected the data if needed.The white matter corrections were mainly targeted at the anatomical areas located below the lateral ventricles (not recognized as white matter by the software) and the circulus Willis area (the blood vessels were excessively falsely recognized as white matter). We did the pial surface corrections on the BrainMask Volume Processing Tool in FreeSurfer, which assessed the excessively recognized parts of the dura and middle cerebral arteries. The control points were set in T1 when the white matter was not fully recognized by FreeSurfer.

To register the fractional anisotropy maps to the anatomical images, we registered the conformed output data (orig.mgz) to an original anatomical dataset (rawavg.mgz) using FreeSurfer’s tkregister2 tool (FreeSurfer data to structural space) and we registered fractional anisotropy maps to the structural space using FMRIB’s Linear Image Registration tool. After these registrations, we concatenated the matrices to form a transformation matrix. This made it possible to transfer the FreeSurfer-calculated volumes and surface structures into the diffusion space.

### Tractography

Tractography was performed separately for both data sets, the one with susceptibility corrections and the one without corrections (Fig. 1). We performed tractography of the corpus callosum by taking the seed regions of interest from the automatic cortical parcellation and labeling the anatomical data. The areas that were included in the seed regions of interest in the corpus callosum were the posterior, mid-posterior, central, mid-anterior and anterior areas. Tracking was restricted using brainstem volume as an avoid mask.

Tracks in the craniocaudal direction were selected using the brainstem as a seed area. The area was selected from the Desikan-Killiany Atlas [31]. Tracking was restricted using masks for the corpus callosum and cerebellum white matter as avoid masks. These craniocaudal tracks mainly represent motor corticospinal pyramid tracks.

We took surface seeds for cingulum tractography from the Desikan-Killiany-Tourville Atlas [33]. Surfaces included in the surface mask were the rostral anterior cingulate, caudal anterior cingulate, posterior cingulate, isthmus cingulate and parahippocampal cortex surface.

We performed tracking bilaterally using the probtrackx2 tool [34, 35]. We did tracing using normal settings, correcting path distribution for the length of the pathways. Using these two data sets, we calculated the fiber length distribution using the fslmaths tool. With the fslstats tool, we saved the histogram using 500 bins. We then transferred the tracts to the diffusion space. The tracts were thresholded and binarized and used as masks. Then we read the fractional anisotropy and mean diffusivity values.

### Statistical analysis

We analyzed the tractography-related parameters using the non-compartmental analysis method. The area under the curve showed a probability density function of the found tracts, which could be seen as the total length of the tract. We used both diffusion metrics and histogram data as continuous variables.

All statistical analyses were performed with R studio 3.5.1 [36]. We used Bland–Altman plots to check the agreement of the two analysis pipelines. The differences between the methods were evaluated using the mean of the difference between methods — in other words, the bias and 1.96 standard deviations above and below the mean difference. We performed the Bland–Altman analysis using the BlandAltmanLeh package. The normality of the bias was tested using the Shapiro–Wilk test. To further evaluate the statistical significance of the bias between the full and partial analysis, we used a paired *t-*test when the data were normally distributed and the Wilcoxon signed rank test when not. A linear regression model was adjusted between the mean values, and we used the bias to analyze whether the bias depended on the mean value of the measured parameter.

## Results

The main result of this study is that there was a significant difference between the susceptibility-corrected and uncorrected pathways. A significant difference was present in both in the diffusion metrics and fiber lengths. The diffusion metrics of the tracts, from both the uncorrected and corrected data, are shown in Table 2.

**Table 2:** Diffusion metrics in uncorrected and corrected data: mean (min–max), standard deviation

Tract	Fractional anisotropy uncorrected	Fractional anisotropy corrected	Mean diffusivity ×10^−3^ (mm^2^/s) uncorrected	Mean diffusivity ×10^−3^ (mm^2^/s) corrected
Corpus callosum	0.341 (0.318–0.365), 0.012	0.339 (0.313–0.365), 0.012	0.996 (0.912–1.004), 0.026	0.968 (0.920–1.020), 0.028
Corticospinal tract	0.346 (0.319–0.369), 0.012	0.344 (0.315–0.369), 0.013	1.045 (0.920–1.151), 0.047	1.071 (0.949–1.181), 0.049
Right cingulum	0.272 (0.252–0.301), 0.012	0.267 (0.250–0.290), 0.011	0.939 (0.900–1.020), 0.030	0.954 (0.911–1.047), 0.034
Left cingulum	0.265 (0.233–0.297), 0.013	0.261 (0.232–0.293), 0.013	0.953 (0.895–1.070), 0.039	0.966 (0.901–1.087), 0.041

In both the fractional anisotropy and mean diffusivity values, the biases between the corrected and uncorrected pathways are significant. The findings were systematically present in all the measured areas. The *P-*values for the corpus callosum, corticospinal tract, right cingulum and left cingulum were ≤0.001 for both diffusion metrics.

**Fig. 2:**
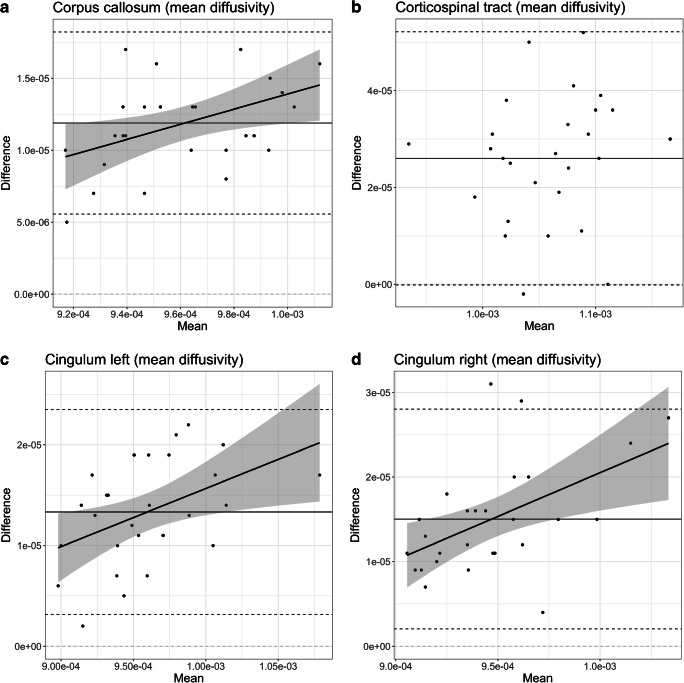
Bland–Altman plots and linear regression models. **a–d** Mean diffusivity values for the corpus callosum (**a**), corticospinal tract (**b**), left cingulum (**c**) and right cingulum (**d**)

**Fig. 3:**
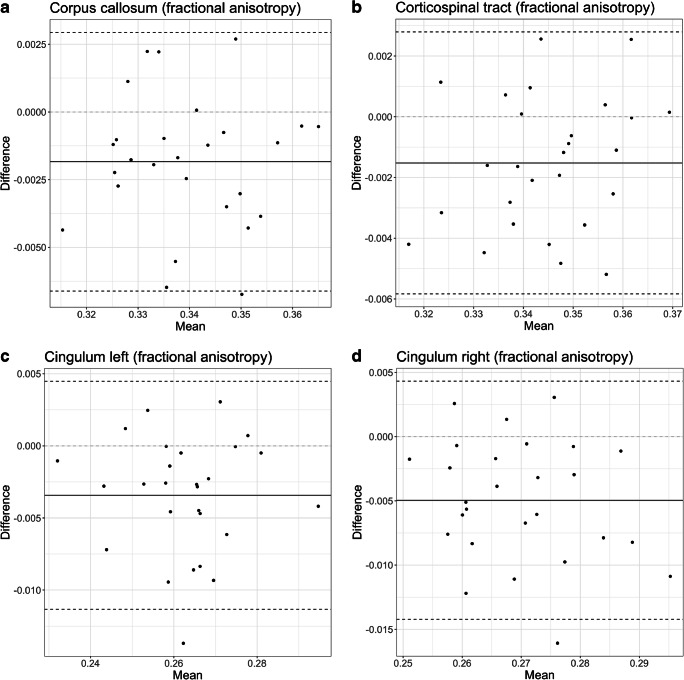
Bland–Altman plots and linear regression models. **a–d** Fractional anisotropy values for the corpus callosum (**a**), corticospinal tract (**b**), left cingulum (**c**) and right cingulum (**d**)

In the mean diffusivity values, a higher mean diffusivity mean value coexisted with a bigger measured bias. This was seen when the linear regression model was adjusted. The model showed a significant positive association between the bias and the mean diffusivity mean value of the analyzed structure. The association was significant in the corpus callosum (*P*=0.02, b=5.251e^−02^, adj.R^2^=0.1665), right cingulum (*P*=0.007, b=1.033e^−01^, adj.R^2^=0.2267) and left cingulum (*P*=0.02, b=5.779e^−02^, adj.R^2^=0.1760). Bland–Altman plots and linear regression models of the mean diffusivity values are shown in Fig. 2. None of the fractional anisotropy values showed significance in linear regression models (Fig. 3), meaning that the bias was not related to the mean value.

**Fig. 4:**
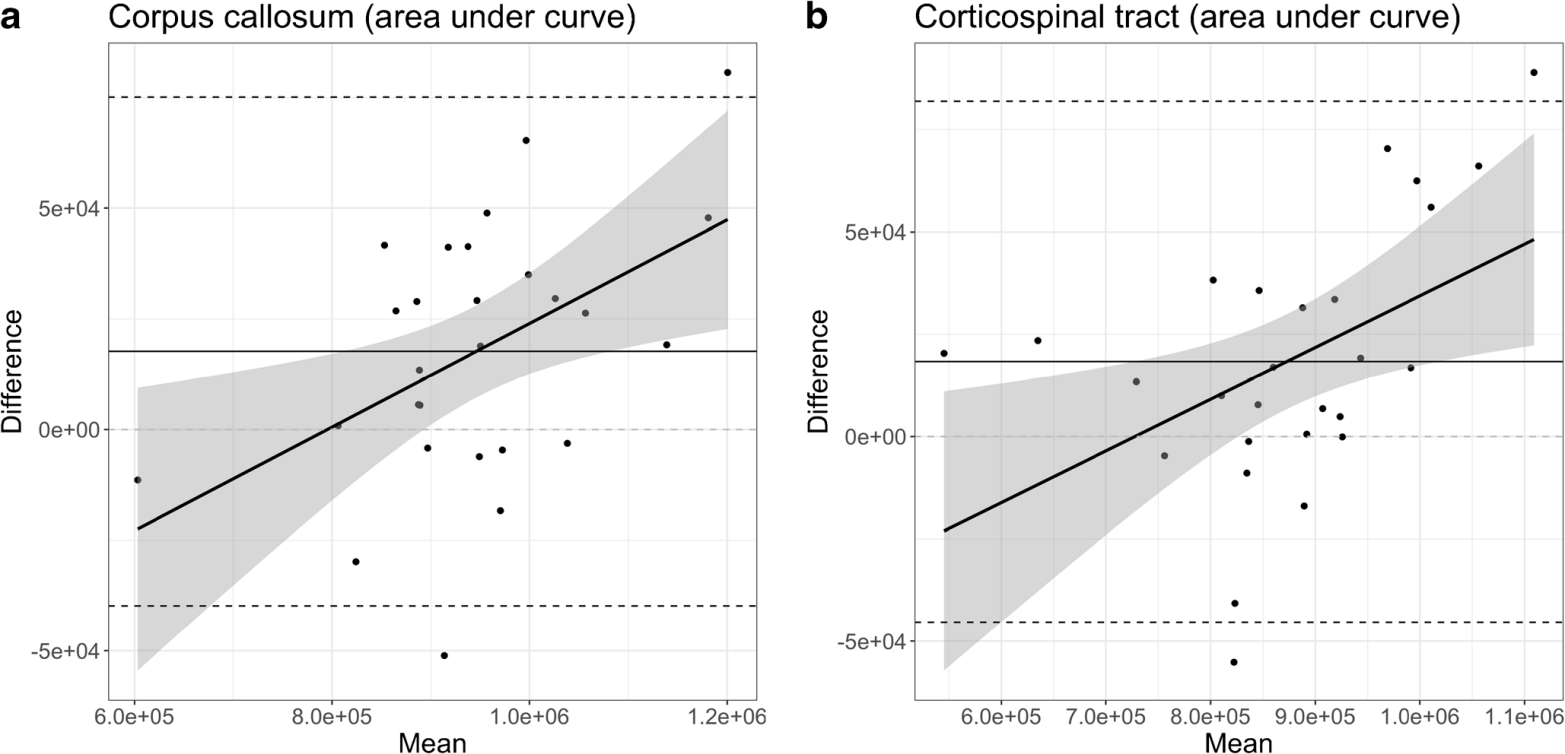
The statistically significant linear regression models of the Bland–Altman plots of the area under the curve values — in other words, total tract length distribution. **a, b** Area under the curve values for the corpus callosum (**a**) and corticospinal tract (**b**)

In tractography analysis, the bias between the corrected and uncorrected analysis pathways reached a statistical significance in the area under curve of corpus callosum (*P*=0.004) and corticospinal tract (*P*=0.007). The tracts appeared longer in the corrected analysis. The adjusted linear regression model showed that the longer the tract, the bigger the bias in tractography, as well. The bias was positively associated to the total length of the tract in two of the analyzed tracts. The effect of the mean area under curve, i.e. the total length, on the bias was statistically significant in the corpus callosum (*P*=0.011, b=1.171e^−01^, adj.R^2^=0.1997) and corticospinal tract (*P*=0.014, b=1.264e^−01^, adj.R^2^=0.1889). The statistically significant linear regression models of the area under curve are shown in Fig. 4.

All biases and their ranges (corrected–uncorrected) between the pathways, the *P-*values of the biases, and the *P-*values and estimates for all regression analyses are shown in Table 3.

**Table 3:** All biases and their range (corrected minus uncorrected) between the pathways, the *P-*values of the biases, and the *P*-values and estimates for all regression analyses

Tract and measure	Bias	Range of bias	Difference *P*	Regression *P*	Estimate
Corpus callosum					
Area under curve	17,700	[−39,800, 72,500]	0.0043	0.0113	0.117
Fractional anisotropy	–1.84e^−3^	[−6.61e^−3^, 2.94e^−3^]	0.0006	0.947	–2.61e^−3^
Mean diffusivity (mm^2^/s)	1.19e^−5^	[0.56e^−5^, 1.82e^−5^]	< 2.2e^−16^	0.0198	5.25e^−2^
Corticospinal tract					
Area under curve	18,300	[−45,300, 82,000]	0.0069	0.0136	0.126
Fractional anisotropy	–1.52e^−3^	[−5.84e^−3^, 2.79e^−3^]	0.0014	0.258	39.1e^−3^
Mean diffusivity (mm^2^/s)	2.60e^−5^	[−0.01e^−5^, 5.21e^−5^]	1.63e^−10^	0.498	3.78e^−2^
Right cingulum					
Area under curve	2,240	[−48,400, 52,900]	0.6617	0.845	8.41e^−3^
Fractional anisotropy	–4.95e^−3^	[−14.2e^−3^, 4.31e^−3^]	1.04e^−5^	0.286	−91.6e^−3^
Mean diffusivity (mm^2^/s)	1.50e^−5^	[0.20e^−5^, 2.80e^−5^]	5.83e^−6^	0.007	10.3e^−2^
Left cingulum					
Area under curve	2,730	[−59,700, 65,200]	0.8593	0.0674	0.090
Fractional anisotropy	−3.43e^−3^	[−11.3e^−3^, 4.47e^−3^]	0.0002	0.868	−10.7e^−3^
Mean diffusivity (mm^2^/s)	1.33e^−5^	[0.32e^−5^, 2.35e^−5^]	3.80e^−13^	0.0169	5.78e^−2^

## Discussion

This study shows that susceptibility correction leads to a statistically significant difference in diffusion metrics and tract lengths in three large, differently oriented tracts — the corpus callosum, cingulum and corticospinal tract — when compared to uncorrected data. In this study, the correction resulted in lower fractional anisotropy values, higher mean diffusivity values and longer tracts in the corrected pathway than the uncorrected pathway.

### Diffusion metrics

Susceptibility distortion correction has been shown to cause a significant reduction in whole-brain white matter fractional anisotropy [10]. The anatomical location has also been shown to affect the quantity of distortion [9, 37]. The highest variation in fractional anisotropy was seen near the known high susceptibility locations, for example in areas near the sphenoid sinus and temporal petrous bone [17]. Taylor et al. [38] demonstrated a transition of whole-brain fractional anisotropy distribution toward lower values in a sample of six children, which is in line with the adult studies.

The biases between the corrected and uncorrected analyses in this study are small compared to the actual measured diffusion metrics. The ranges for the limits of agreement of the biases were close to the standard deviation values of the measured values. The biases were also small compared to the variation between the measured values in this study and the previous studies of Carper et al. [39], Epstein et al. [40], Rocca et al. [41] and Vulser et al. [42]. The diffusion tensor imaging metrics of previous studies in this age group are presented in Table 4 [39–42].

**Table 4:** The diffusion tensor metrics fractional anisotropy (FA) and mean diffusivity (MD) in mm^2^/s (standard deviation [SD]), based on previously published combined samples of children and adolescents

Study	Method	Scanner	*n*, age in years (SD)	Metrics	Corpus callosum	Corticospinal tract, right	Corticospinal tract, left	Cingulum, right	Cingulum, left
Carper et al. [39]	Probabilistic tractography	GE Discovery 3.0T	36, 12.8 (2.4)	FA	Not measured	0.44 (0.02)	0.43 (0.02)	Not measured	Not measured
				MD	Not measured	0.80 (0.03)	0.80 (0.03)	Not measured	Not measured
Epstein et al. [40]	Probabilistic tractography	Siemens Trio 3.0T	55, 16.5 (2.6)	FA	Not measured	0.59 (0.02)	0.60 (0.02)	0.44 (0.03)	0.46 (0.03)
				MD	Not measured	Not measured	Not measured	Not measured	Not measured
Rocca et al. [41]^a^	Tract-based spatial statistics	GE LX 1.5T	13, 12.2 (2.7)	FA	0.60 (0.02)	0.63 (0.03)	0.63 (0.02)	Not measured	Not measured
				MD	0.81 (0.03)	0.76 (0.04)	0.75 (0.03)	Not measured	Not measured
Rocca et al. [41]^b^	Tract-based spatial statistics	Philips Intera 3.0T	18, 12.9 (2.7)	FA	0.59 (0.02)	0.66 (0.02)	0.65 (0.02)	Not measured	Not measured
				MD	0.77 (0.02)	0.77 (0.02)	0.77 (0.02)	Not measured	Not measured
Vulser et al. [42]	Tract-based spatial statistics	14 different 3.0T scanners^c^	336, 14.4 (0.4)	FA	Not measured	Not measured	Not measured	0.36 (0.04)	0.38 (0.04)
				MD	Not measured	Not measured	Not measured	0.80 (0.05)	0.81 (0.05)

^a,b^The study by Rocca et al. [41] included two different study centres, The Hospital for Sick Children, Toronto, Canada (marked with a) and Ospedale San Raffaelem ‘Vita-Salute’ San Raffaele University, Milan, Italy (marked with b)

^c^This study is a part of Imagen Consortium (www.imagen-europe.com); all manufacturers (GE, Philips and Siemens) were used

The fractional anisotropy values measured in this study were lower and mean diffusivity values higher than those reported earlier, but the metrics were not directly comparable. The studies conducted by Carper et al. [39] and Epstein et al. [40] were done based on a combined sample of children and adolescents ages 7–18 years and 10−23 years, respectively, which might affect the metrics [3]. The age range for the study subjects of Rocca et al. [41] was 12−13, similar to this study, but the seed region was different for the corticospinal tract. The seed region for the corpus callosum was similar, but the tract was thresholded using a stricter limit [41]. The corpus callosum tract in the present study is thereby more likely to also include the outer, less homogeneous areas of the tract. The partial volume effect and looser axonal organization tend to lower the fractional anisotropy value in these outer areas. Also, the tractography method and the scanners varied among the previous studies, presented in Table 4. It is noteworthy that not all of these studies corrected their data for susceptibility distortions nor stated the method used when doing so.

Our study showed that the bias was associated with the measured parameter in certain tracts. In mean diffusivity measurements, the association between the bias and the mean was seen bilaterally in the cingulum, as well as in the corpus callosum. However, the effect was minor and likely to be explained by the increase in the mean value. For fractional anisotropies, no associations were found. The bias of the mean diffusivity values was systematic and thereby we assume that it unlikely is coincidental.

### Tract length

In this study, the susceptibility-corrected data showed a positive bias between the analysis pathways when comparing the total tract length. The bias was statistically significant in tracts originating from the corticospinal tract and corpus callosum. The left or right cingulum showed no total tract length bias between the analysis pathways. One could speculate that this finding reflects the effects of anatomical location. The effect size of the susceptibility correction was the largest in the peripheral areas of the brain, while the cingulum was centrally located.

A few reports of susceptibility-related pilot tractography studies have looked at adults. Embleton et al. [16] showed that in the temporal areas, the distortion correction affects tractography. They stated that before the correction, the most significant problems tended to occur near the third and fourth ventricles, where the susceptibility-related artifacts are generally severe [16]. Our study showed that the effect is also visible in other lobes.

We found a significant difference in tract lengths between the corrected and uncorrected pathways. Many of the studies assessing these methodological issues were done using phantoms or adult subjects [9, 43, 44]. Previously, Irfanoglu et al. [18] found that susceptibility correction affects the principal eigenvector orientation, tract continuity, tract length and the probability of reaching anatomically correct cortical regions, left–right symmetry, the number of tracts and their spatial variance. Taylor et al. [38] showed that the sensitivity and specificity of group tractography results also change in retrospective processing. The present study shows that the mean tract length in the corpus callosum and corticospinal tract is positively associated with the bias, though this association is relatively minor and likely to be associated with the increase in the mean value.

### Implementation and impact

Diffusion imaging as a method is a widely used when studying adolescents, but methodological studies in this age group are scarce [1–3, 24, 38, 43, 45]. The mean diffusivity values are higher in children than in adults [3], which further highlights the importance of using the correction at this age. The dental braces, which can cause susceptibility issues, are also seen more frequently in adolescents [46, 47].

Irfanoglu et al. [10] raised discussion about the importance of susceptibility correction in different settings. The present study is in line with the previous literature and highlights the need for the systematic use of correction methods. Susceptibility correction has an impact on the planning of multi-site collaborative studies, comparing newer and older research data, but also when comparing clinical data to previously published literature. In addition, the correction method and scanner used might affect the results and this should be accounted for in the planning phase of the study [18, 37].

## Conclusion

Susceptibility correction choices affect the diffusion metrics and tract lengths. Correction should be used systematically to facilitate comparison among studies.
